# Advancing discovery in hearing research via biologist-friendly access to multi-omic data

**DOI:** 10.1007/s00439-022-02445-w

**Published:** 2022-03-02

**Authors:** Ronna Hertzano, Anup Mahurkar

**Affiliations:** 1 Department of Otorhinolaryngology Head and Neck Surgery, University of Maryland School of Medicine, Baltimore, MD 21201 USA; 2 Department of Anatomy and Neurobiology, University of Maryland School of Medicine, Baltimore, MD 21201 USA; 3 Institute for Genome Sciences, University of Maryland School of Medicine, Baltimore, MD 21201 USA

## Abstract

High-throughput cell type-specific multi-omic analyses have advanced our understanding of inner ear biology in an unprecedented way. The full benefit of these data, however, is reached from their re-use. Successful re-use of data requires identifying the natural users and ensuring proper data democratization and federation for their seamless and meaningful access. Here we discuss universal challenges in access and re-use of multi-omic data, possible solutions, and introduce the gEAR (the gene Expression Analysis Resource, umgear.org)—a tool for multi-omic data visualization, sharing and access for the ear field.

## Introduction

Omics data generation and analysis has undergone rapid expansion since the publication of the human and mouse genomes barely two decades ago (Craig Venter et al. 2001; Waterston et al. 2002). Since then, technological advances have improved the speed, throughput, accuracy, and affordability of these technologies. In addition, advancements in the last few years enable many of these interrogations to be performed at the resolution of single cells allowing us to understand the spatial and temporal dynamics at a very high (cell-level) resolution (Longo et al. 2021). These advances have been widely adopted in the ear field with a growing number of datasets generated and published on an annual basis. Disabling hearing loss, which affects 1:1000 newborns and over 50% of the population over 70, results from mutations in over 150 genes distributed in their expression across the different cell types of the mammalian inner ear (Kremer 2021). Cell type-specific omics have advanced our understanding of the inner ear cell types (Burns et al. 2015; Korrapati et al. 2019; Wilkerson et al. 2021), identified critical regulators of cell fate (Hertzano et al. 2011; Elkon et al. 2015; Wiwatpanit et al. 2018; Chessum et al. 2018; Matern et al. 2020), and uncovered some of the challenges in hair cell regeneration in mammals (Menendez et al. 2020; Tao et al. 2021). However, the value of these and many additional datasets exceeds the discrete scientific findings reported in the published literature. The full value of these data is reached by re-use of the data which requires the ability to find, access, visualize and analyze the data by potential users.

## Availability and access to multi-omic data

Multi-omic data serve as the basis for discovery and are usually published in conjunction to peer-reviewed manuscripts. While the manuscripts highlight key findings, and may offer pertinent gene lists as attached tables, by convention, all the data, raw as well as processed, are deposited in repositories, such as the NCBI’s Sequence Read Archive (SRA) (Leinonen et al. 2011b) and EMBL–EBI’s European Nucleotide Archive (ENA) (Leinonen et al. 2011a) for raw sequence data, and NCBI’s Gene Expression Omnibus (GEO) (Clough and Barrett 2016) and EMBL–EBI’s ArrayExpress (Athar et al. 2019), European Variant Archive (EVA; https://www.ebi.ac.uk/eva/) for gene expression and variant data. It is the availability and subsequent reuse of these data by other users for new discoveries that increases their value. With the increased prevalence of multi-omic data, stakeholders representing a diverse range of users have established guidelines for findability and ease of reuse through improved FAIR-ness: Findability, Accessibility, Interoperability and Reuse (Wilkinson et al. 2016) and TRUST-worthiness: Transparency, Responsibility, User focus, Sustainability and Technology of the data (Lin et al. 2020). The adoption and adherence to such principles is critical as the volume and complexity of the data continue to increase, and their access relies on standardized computational approaches.

## Data democratization and federation

Data democratization is the process of making digital data accessible to the “average user”. One of the goals of data democratization should be the empowerment of users to find and analyze the data without additional (expert) help. This is a universal concept that applies across disciplines (from business to medicine) and is also relevant to multi-omic data. For successful data democratization, we must consider the definition of the average user. In the case of multi-omic data, there are two distinct personas. The first, and less prevalent, is the bioinformatician or computational biologist. This user is often interested in the raw data for re-use and analysis, although often times uses analyzed data such as expression matrices or variant calls. The need to download the data for reprocessing and analysis is acceptable and trivial for such users. The second is the biologist that is familiar with concepts of data analysis and bioinformatics but is not computationally trained. Furthermore, the biologist often does not have access to the necessary infrastructure to work with raw data or analyzed matrices. For the biologist, who is the most prevalent ‘consumer’ of these data, it is important for the data to be presented in an accessible format that allows seamless and rapid analysis, visualization, and ability to share their data—without requiring its download. Equally important is the speed to find and access data, and ability to compare across datasets.

Previously, users could expect that there would be a single repository to access sequence-based omic data. It is now impractical to centralize the vast number of data sets being generated by the research community. To overcome distribution of the resources across continents and repositories, several efforts are underway to federate the data. Data federation deals with generating virtual meta-databases that allow the interconnection of distributed databases so users can find data across these repositories through a centralized mechanism. One such attempt, the Global Alliance for Genomics and Health (GA4GH; https://www.ga4gh.org/), takes the approach of defining data and metadata standards, and application programming interfaces that are adopted by multiple data repositories, which allow users to build tools to discover, interrogate, and download data from distributed repositories.

In addition to the challenges of distributed data, another challenge is the ability to compare or combine data that are generated or analyzed using disparate systems and tools. One way to address this challenge is to generate and process data using the same technology and tools. Examples of large international consortia that take this approach include the 1000 Genomes Project (https://www.internationalgenome.org/), The Cancer Genome Atlas (TCGA; https://www.cancer.gov/tcga), the Encyclopedia of DNA elements (ENCODE; https://www.encodeproject.org/) (Davis et al. 2018), EpigenomeRoadmap (http://www.roadmapepigenomics.org/), International Cancer Genome Consortium (ICGC; https://dcc.icgc.org/), and the Genotype-Tissue Expression portal (GTEx; https://gtexportal.org/). Another approach is to develop resources, where existing data are reanalyzed using same tools and technologies. One such example is the Recount2 project, where the group reanalyzed all the RNAseq data that was available in public repositories in 2015 to allow comparison of data across experiments and projects (Collado-Torres et al. 2017). A more recent approach is to bring data and computational pipelines together in a shared environment or ecosystem to enable users to reanalyze data easily as necessary. The Broad Institute’s Terra computational platform is one such resource, where data from multiple projects, and common tools and pipelines are available for data reprocessing as needed (Perkel 2022).

## A variety of tools for browsing of multi-omic data

With the popularization of multi-omics data as a workhorse for discovery in biological sciences, an increasing number of analysis and visualization tools have become available. These can be broadly divided into three groups. The first group includes general purpose analysis and visualization tools including Bioconductor packages (Huber et al. 2015), Docker containers, or Jupyter notebooks that are geared towards informaticians or informatics savvy users. The second group include tools or repositories developed to disseminate data that are focused on a specific project, disease, or datatype. These are divided into ‘closed’ and ‘open’ resources. That is resources where all the data are generated by a specific consortium/repository (e.g., the data portals for TCGA and ICGC for cancer research, or the Human Microbiome Project data portal (https://portal.hmpdacc.org/)) and open, where in addition to data generated by the portal managers, data from a specific field are collected and curated for the benefit of a specific research community. Examples of these include The Accelerating Medicines Partnership Program for Alzheimer's Disease (AMP–AD; https://adknowledgeportal.synapse.org/) directed towards the Alzheimer’s Disease community (Greenwood et al. 2020), the gene Expression Analysis Resource (gEAR; https://umgear.org/) directed towards the hearing research community (Orvis et al. 2021), or the Neuroscience Multi-Omic Analytics (NeMO Analytics; https://nemoanalytics.org/) geared towards neuroscience community (BRAIN Initiative Cell Census Network 2021). Within these resources, some portals or tools are directed towards informaticians, while others are geared towards biologists such as GTEx expression and expression Quantitative Trait Loci (eQTL) browser, or the Xena Functional Genomics Explorer (https://xenabrowser.net/) for TCGA data. The third group include general purpose visualization tools, where users can upload or view their own data in specific tools such as University of California Santa Cruz Browser (https://genome.ucsc.edu/) (Haeussler et al. 2019), Broad Institute’s Single Cell Portal (https://singlecell.broadinstitute.org/single_cell), or cellxgene (https://cellxgene.cziscience.com/). Repositories for user-based data upload and analysis, are designed to allow users to upload datasets to the public domain. As the number of datasets in these repositories grow, so does meaningful access to data. However, upload of data still requires some bioinformatics expertise, file formats are not uniform across platforms, and finding the data is often a challenge. Another limitation is the need for fully analyzed data for meaningful browsing. This is particularly challenging as most journals mandate the deposition of the raw but not the analyzed data into the GEO. This presents a particularly important challenge when dealing with single cell-based data, where the main value of the work is in the published analysis.

## The gEAR portal—gene expression analysis resource (umgear.org)

The wealth of multi-omic data generated for the ear field has been uploaded, curated and shared via the gEAR portal (Orvis et al. 2021). The gEAR is designed as a web-based interface for visualization, sharing and analysis of multi-omic data. It is unique in its ability to present numerous datasets across species and modalities, side by side, in one page—enabling the user to meaningfully browse and compare data. It currently displays over 150 public datasets organized in thematic profiles that are categorized based on topic (e.g., development, aging, damage) or by manuscript. A dataset manager allows users to build new dataset collections. A dataset uploader allows users to upload their data and use it in the private domain or share with collaborators, also before it is ready for public release. Short links (permalinks) can be generated and added to manuscript figure legends to allow interactive browsing of published and analyzed datasets by simply clicking on the figure legend. Analysis tools include a tool to compare gene expression across conditions, an elaborate workbench for analysis of single cell data, a tool to build and interrogate gene lists (gene carts) across conditions, and options for data download and export. The resource is keyed by gene symbols and in addition to common ontological annotation, provides specific annotation regarding known involvement of genes in hearing loss in mouse or human, and links to ear-specific resources such as the Deafness Variation Database (Azaiez et al. 2018). The gEAR has become a primary resource for data sharing within the ear field and is cited for data validation, hypothesis generation, and data dissemination. The code, which is open source, has now been used to support other communities, including the BRAIN initiative via NeMO Analytics and the infectious diseases research community via the Genomics Centers for Infectious Diseases (GCID; https://gcid.umgear.org/) at University of Maryland.

## Closing remarks

The transformative impact that omics technological advancements have on biological sciences and medicine cannot be overestimated. With these advancements, however, come a host of challenges. Size of files, access to data, appropriate form of data storage, data annotation and appropriate metadata for experiments to name a few. While data democratization is progressing, better guidelines for data sharing with publications are necessary. Furthermore, training for researchers in health sciences to improve access to multi-omic data is also needed. In parallel, solutions have been developed, and need continued development to provide more meaningful access to multi-omic data for biologists that are not informatics trained. The gEAR is an important example of this approach and provides meaningful access to multi-omic data for a specific research community, the hearing research community. However, such efforts require extensive investment. Should such resources be managed by the funding agencies, such as the NIH, to provide democratized and possibly federated access to multi-omic data across all fields? Should funding be contingent on better data sharing and annotation? Finally, can we arrive at common standards for files, and the all-important metadata associated with the samples that are used to generate omic data? These are all important questions that if addressed collectively, but primarily by the funding agencies, could propel discovery via the broad use and reuse of multi-omic data across disciplines.

## Data Availability

Not applicable.
